# Power-Law Input-Output Transfer Functions Explain the Contrast-Response and Tuning Properties of Neurons in Visual Cortex

**DOI:** 10.1371/journal.pcbi.1001078

**Published:** 2011-02-24

**Authors:** Erez Persi, David Hansel, Lionel Nowak, Pascal Barone, Carl van Vreeswijk

**Affiliations:** 1 Laboratoire de Neurophysique et Physiologie, Université Paris Descartes, Paris, France; 2 Interdisciplinary Center for Neural Computation, The Hebrew University, Jerusalem, Israel; 3 Cerco, Université Toulouse 3, CNRS, Toulouse, France; Gatsby Computational Neuroscience Unit, UCL, United Kingdom

## Abstract

We develop a unified model accounting simultaneously for the contrast invariance of the width of the orientation tuning curves (OT) and for the sigmoidal shape of the contrast response function (CRF) of neurons in the primary visual cortex (V1). We determine analytically the conditions for the structure of the afferent LGN and recurrent V1 inputs that lead to these properties for a hypercolumn composed of rate based neurons with a power-law transfer function. We investigate what are the relative contributions of single neuron and network properties in shaping the OT and the CRF. We test these results with numerical simulations of a network of conductance-based model (CBM) neurons and we demonstrate that they are valid and more robust here than in the rate model. The results indicate that because of the acceleration in the transfer function, described here by a power-law, the orientation tuning curves of V1 neurons are more tuned, and their CRF is steeper than those of their inputs. Last, we show that it is possible to account for the diversity in the measured CRFs by introducing heterogeneities either in single neuron properties or in the input to the neurons. We show how correlations among the parameters that characterize the CRF depend on these sources of heterogeneities. Comparison with experimental data suggests that both sources contribute nearly equally to the diversity of CRF shapes observed in V1 neurons.

## Author Summary

Both the response and membrane potential of neurons in the primary visual cortex (V1) are selective to the orientation of elongated stimuli. The widths of the tuning curves, which characterize this selectivity, hardly depend on stimulus contrast whereas their amplitude does. The contrast dependence of this amplitude, the contrast response function (CRF), has a sigmoidal shape. Saturation of the spike response is substantially lower than the neurons' maximal firing rate. These well established facts constrain the possible mechanisms for orientation selectivity in V1. Furthermore, the single neuron CRFs in V1 display a broad diversity in their shape. This adds other constraints. Many theoretical works have tried to elaborate mechanisms of orientation selectivity that are compatible with the contrast invariant tuning widths. However, these mechanisms are usually incompatible with sigmoidal CRFs. We propose a mechanism which accounts simultaneously for contrast invariant tuning width for both rate and voltage response and for the shape and diversity of the CRFs. This mechanism relies on the interplay between power-law frequency-current transfer functions of single neurons, as measured in vivo in cortex, and on the recurrent interactions in the cortical circuit.

## Introduction

The dependence of the neuronal response amplitude on stimulus contrast, the contrast-response function (CRF), typically displays a sigmoidal shape in the visual cortex: it accelerates at low contrast and saturates at high contrast [1]–[8]. This major nonlinearity appears to be accentuated in cortex, as ganglion cells in the retina and relay cells in the LGN saturate at higher contrast and show shallower slopes [3], [7], [9]–[15]. In the extreme, some parvocellular neurons in primate LGN display a quasi-linear contrast-response function [13], [14], [16].

A large fraction of neurons in the primary visual cortex (V1) respond in a manner that is selective to the stimulus orientation [17], [18]. The dependence of the spike rate on stimulus orientation (the orientation tuning curve) is well described by a Gaussian whose amplitude varies substantially with stimulus contrast. Although this might be less true in primate [19], [20], it has been shown in carnivore and rodents that the width of the tuning curves does not change when the contrast is modified [2], [7], [15], [21]–[25] This property is referred to as “contrast invariance” of orientation tuning. The membrane potential response of cortical neurons displays an orientation tuning width that is typically 1.5 times larger than that of the spiking response [15], [26]–[30]. This tuning width is also contrast invariant [23]. These contrast-invariant properties constitute strong constraints for understanding the mechanisms underlying the response of V1 neurons to visual stimuli.

The models that have been proposed to explain orientation selectivity in V1 can be broadly classified in two groups (reviewed in [31], [32]): feedforward models, in which orientation selectivity emerges mainly from the spatial arrangement of ON and OFF receptive fields of the LGN cells that form the input to V1 neurons, and recurrent network models in which the orientation selectivity emerges mainly from the recurrent connectivity within V1. Both classes of models have limitations. Although recurrent models can account for contrast invariance in the spiking response [33]–[37], they appear incompatible with the fact that V1 recurrent inputs seem to have, at best, a very weak effect on the voltage tuning width [38], [39]. Recurrent models have further been questioned given the peculiar responses they generate in the presence of pairs of oriented contours [40] and given strong interaction between spatial frequency and orientation selectivity [36]. Furthermore, in contradiction to the experimental results, the response of neurons in such models either display contrast invariance of orientation selectivity, or CRFs saturation, but not both simultaneously [41], [42].

The feedforward model, in its original formulation [17], cannot account simultaneously for the fact that orientation tuning of the spike response is sharper than the tuning of the voltage and for the contrast invariance of the spike response tuning width. Nevertheless, including feedforward anti-phase inhibition [43] or broadly tuned inhibition [44], [45] in the feedforward model permits contrast-invariance of orientation tuning in the membrane potential response. Anderson et al. [23] further showed that contrast invariance of orientation tuning for the spiking response, in addition to that of the membrane potential response, can be achieved in the feedforward model if membrane potential fluctuations (“synaptic noise”) are taken into account. This is because these fluctuations smooth the threshold non-linearity [15], [46], [47]. This smoothing effectively transforms the transfer function of the neurons to a power-law voltage-rate relationship. This is exactly what is needed to obtain contrast invariance for both the voltage and the firing rate, provided membrane potential fluctuations amplitude scales with contrast [15]. However, the feedforward model may account for sigmoidal CRFs only if the LGN input saturates sufficiently strongly. Yet the contrast at which saturation occurs in V1 is lower than for the LGN input. This implies that additional mechanisms are required to account for the co-occurrence of contrast-invariant orientation tuning and of CRFs typical of V1 neurons.

Some of the models used to examine the mechanisms responsible for the saturation of the CRF also display contrast-invariant stimulus selectivity. In the “normalization model” [48]–[51], saturation results from feedback shunting inhibition from a pool of inhibitory neurons. Because this pooling includes inhibitory neurons with a wide range of preferences, this model also accounts for cross-orientation inhibition as well as for contrast-invariance of orientation tuning. However, this model has been questioned due to membrane time constants requirements [32].

Alternatively, synaptic depression has been proposed as one mechanism to explain saturation at high contrast [52], [53]. In these models however, contrast-invariance of orientation tuning does not depend on synaptic depression but depends on the push-pull arrangement of inhibition and excitation, as in the model proposed by Troyer et al. [43]. In another recent model, Banitt et al. [54] examined how contrast-invariance of orientation tuning may depend on thalamocortical synaptic depression, but they did not explore the mechanisms underlying contrast saturation. Models based on synaptic depression are able to explain not only the static properties of the behavior of V1 neurons, but also dynamical aspects, such as contrast-dependent phase advances and frequency-dependent contrast saturation. Nevertheless, recent experimental studies showed that synaptic depression in the thalamocortical pathway may be rather weak *in vivo*, especially in the presence of spontaneous activity that generates a steady state of synaptic depression [55]–[57].

Thus the question is: can one formulate, without resorting to synaptic depression, a model in which cortical neurons display contrast-invariant tuning-width for both membrane potential and spike responses, as in the feedforward model in the presence of synaptic noise, while at the same time intracortical interactions induce a saturation of the CRF of cortical neurons at lower contrast than their LGN afferents ? To examine this question, we investigated a rate model of a hypercolumn in the visual cortex with neurons whose transfer function nonlinearity was described by a power-law. This allowed us to find conditions for getting both a sigmoidally shaped CRF and contrast invariant orientation tuning width when both feedforward and feedback inputs were included.

We then tested whether our results hold in a less idealized network model made of conductance-based (CBM) neurons. Using numerical simulations in this later model, we investigated the robustness of the results obtained in our rate model. We analyzed the respective contributions of the feedforward input, of the recurrent intra-cortical input, and of neuronal intrinsic properties in shaping the CRF. In particular, we studied the differences between inhibitory and excitatory neurons, and how these relate to differences in their intrinsic properties.

Finally, we explored possible explanations for the broad diversity of CRFs shapes observed in V1 neurons: although typically sigmoidal, CRFs are characterized by parameter values that vary widely at the population level [1], [3], [8]. For this purpose, we compared the predictions from our model with experimental data obtained in area V1 of the marmoset monkey. Our results suggest that substantial heterogeneities in the intrinsic properties of the neurons as well as heterogeneities in the CRFs of LGN neurons are required to account for the diversity of CRFs shapes observed in the primary visual cortex.

Part of this work has been presented at the 34th and 36th annual meeting of the Society for Neuroscience (San-Diego, Oct 2004; Atlanta, Oct 2006).

## Results

### The tuning curves of neurons in the hypercolumn rate model

#### Conditions for an exact contrast-invariance of the orientation tuning width

Our model consists of 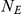 excitatory (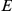) and 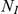 inhibitory (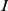) rate units with a power law input-output transfer function. Neuron 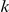 in population 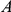 (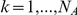, 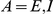) is characterized by its preferred orientation (PO), 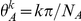. The strength of the synaptic connection between neurons 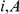 and 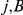 depends on the difference in their preferred orientation, 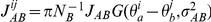, where 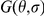 is the 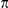-periodic Gaussian with widths 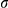, 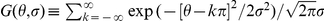. In addition to the recurrent inputs from the network, neuron 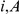 receives a tuned input 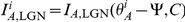, where 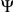 is the stimulus orientation and 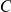 the stimulus contrast. We assume 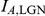 to have a Gaussian shape, 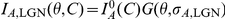, where 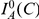 is the visually input for a stimulus at the preferred orientation of the neuron. The latter input represents the total input resulting from the combination of the thalamic excitation to V1 and an untuned cortical feedforward inhibition which cancels the untuned part of the LGN excitation (see Discussion).

In the following, we assume that 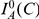 varies logarithmically with contrast (in % of the maximal contrast, C between 0 and 100): 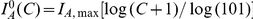. Note that the tuning width of this input does not depend on contrast. More details on the model are given in the Methods Section.

In the absence of recurrent interactions in the network, 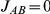, the response of neuron 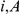 to a stimulus is
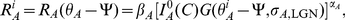
(1)where 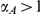 is the exponent of the power-law transfer function of the neuron. If 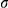 is sufficiently small compared to 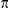, 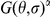 is, to a very good approximation, proportional to 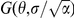. Hence the steady state firing rate satisfies
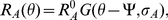
(2)with
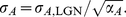
(3)As a result, the output tuning width is contrast invariant and is sharper than the tuning width of the LGN input by a factor 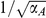.

When the neurons interact, the tuning width of their responses does depend on the contrast, unless some specific conditions, concerning the range of the interactions and the tuning width of the LGN input, are met. To derive these conditions we make the *Ansatz* that 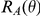 is a periodic Gaussian with standard-deviation 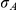: 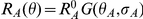. In the large 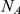 limit the feedback input is 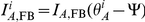, where 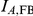 is given by
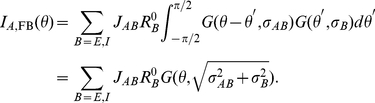
(4)Here we have used the fact that the convolution of two periodic Gaussians is a periodic Gaussian whose variance is the sum of their variances.

Consistency with this *Ansatz* requires that the total input 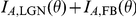 is also a Gaussian. This happens only if
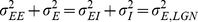
(5)and
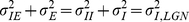
(6)It should be noted that, if these conditions are satisfied, the recurrent interactions do not contribute to the sharpening of the output tuning. Consequently, the output tuning width of the neurons, which is still given by Equation (3), is still contrast invariant.

These conditions can be rewritten as
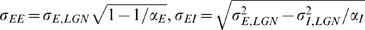
(7)

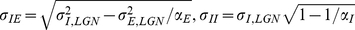
(8)In particular, the assumption that 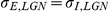 and 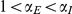, implies that 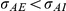, i.e., that the inhibitory projections are broader than the excitatory ones. On the other hand, if the input tuning for the inhibitory cells is broader than for the excitatory ones, 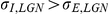, we may obtain that 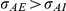, i. e., that the excitatory feedback tuning is broader than the inhibitory one, even if 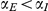. When Eqns. (8) are satisfied, the width of the output tuning curves does not depend on the interactions, but their amplitude does.

Inserting Eqn. (4) into Eqn. (1) one finds that 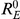 and 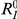 are determined by the self-consistency equations
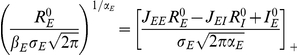


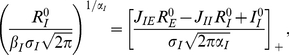
(9)where 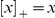 for 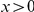 and 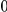 for 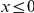.

**Figure 1:**
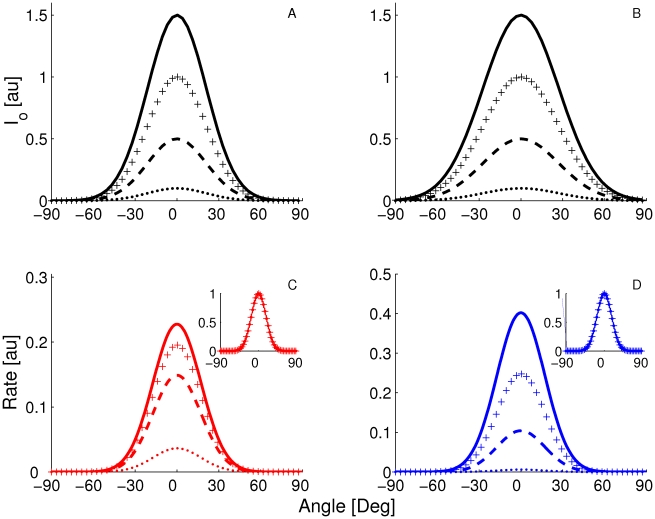
Tuning curves in the rate model when conditions **Eqns. (7**, **8)** are satisfied. A: LGN inputs to the excitatory neurons. B: LGN inputs to the inhibitory neurons for different 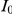. C: tuning curves of the output for these inputs for excitatory cells. D: tuning curves of the output for inhibitory cells. Solid line: 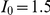, pluses: 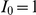, dashed line: 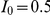, dotted line: 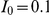. Insets: tuning curves are normalized to their peaks showing that the tuning width is exactly contrast-invariant. Parameters: 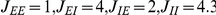, 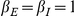, 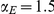, 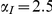, and 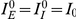.

Equations (9) have been derived under the assumption that the tuning width of the LGN input is much smaller than 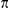. In Fig. 1, we compare the results derived from these equations with the numerical simulations of the dynamics of the model, for a network with 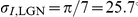, 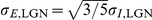 and 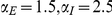. This implies that 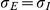. The coupling strengths are such that the maximal firing rate is substantially larger in inhibitory neurons in comparison to excitatory neurons. With these parameters, the width of the output tuning curves is close to 15 degrees, on the order of experimentally reported values for V1 neurons. Fig. 1 shows the input (top) and output (bottom) of the excitatory and inhibitory populations from simulations. The output is narrower than the input and its width is contrast-invariant (insets in the bottom panel). We verified that the simulation results are in excellent agreement with Eqns. (9).

In Supporting Text S1 we determine the stability of the steady state against arbitrarily small perturbations. These calculations show that, in general, the least stable mode corresponds to a modulation in the amplitude of the response without change in the shape of the orientation tuning curve: 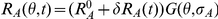. The stability of this mode depends of the ratio between the excitatory and inhibitory time constants, 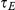 and 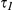 respectively. If 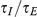 becomes too large, the steady state looses its stability. However, for reasonable values of the network parameters, the restriction on the inhibitory time constant is very weak. For the parameters we have used, the network is stable for 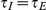.

#### The shape of the contrast response function

The contrast response function of a neuron for a stimulus at its preferred orientation can be computed by solving the self-consistent equations, Eqns. (9). Let us first consider low contrast stimuli, i.e. 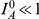. In this regime, the output firing rate is small. Since 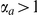, 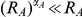 where 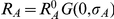 is the firing rate of neurons whose preferred orientation coincides with the stimulus orientation. One finds easily that in this limit the rate responses are, to leading order, proportional to the LGN input, 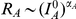. Therefore, for low contrasts there is an acceleration in the response, which depends mostly on the exponent 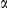 of the neuron's transfer function. The larger 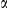 is, the steeper the CRF at low contrast is.

**Figure 2:**
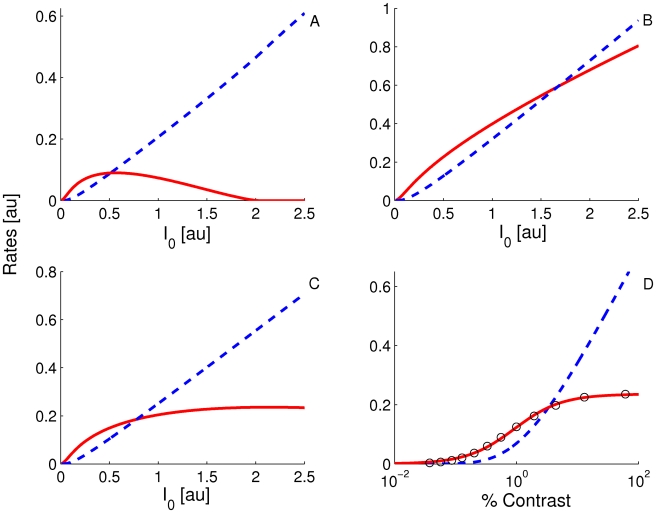
Firing rate vs. LGN input in the rate model. A–C: solutions of Eqn. (9), for the excitatory (solid line) and inhibitory (dashed line) neurons. The parameters, except for 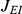, are as in Fig. 1. A: inequality (10) is satisfied (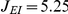). B: inequality (10) is not satisfied. (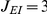). C: inequality (10) is barely satisfied. (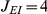). D: activity plotted against contrast assuming 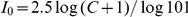. Parameters as in C. Circles: excitatory neurons. Solid line: best fit H-ratio function. Parameters of the fit are 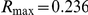, 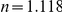, 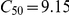 Dashed line: inhibitory neuron's response.

The behavior of the response for large inputs depends on the strength of inhibition between inhibitory neurons, compared to the strength of inhibition they exert on excitatory neurons. Assuming an equal LGN input on both populations, 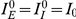, Eqns. (9) indicates that if
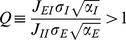
(10)

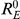
 vanishes for sufficiently large 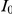. Hence, in that case, the activity of the excitatory population varies non-monotonically with the LGN input whereas the activity of the inhibitory neurons increases (Fig. 2, A–C). This is because the inhibitory neurons receive an input from the LGN. Moreover, the weaker the mutual inhibition between inhibitory neurons, the sharper their firing rates increase with the LGN input 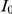. As a consequence, if the inhibition on the excitatory neurons is sufficiently strong, it can suppress the activity of the excitatory populations at large LGN input. An example of this situation is shown in Fig. 2A.

If the inequality (10) is not satisfied, the inhibitory population cannot suppress the activity of the excitatory one. As a consequence, the activities of both populations increase monotonically with no bound with 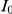. Fig. 2B corresponds to this situation.

The ratio, 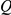, controls the position of the maximum of 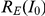 as well as the shape of this curve around it. In Fig. 2C, Q has been chosen so that the curve is very flat around the maximum.

These results imply that three qualitatively different shapes can be found for 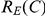 and 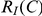 as a function of the contrast, 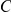. For sufficiently large 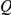, the CRF increases at low contrast but decreases at large contrast. If 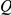 is sufficiently small, so that 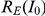 increases monotonically with 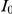, the CRF as well as its derivative increase with the contrast. In some intermediate range of 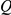, the CRF is still increasing with C, but it displays an inflexion point beyond which it tends to saturate. This happens when 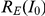 is non-monotonous, with a maximum located at a value of 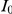 larger than 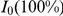. An example of such a saturating CRF is displayed in Fig. 2D. The solid line corresponds to a fit with an H-ratio function (see Methods) which is very good in the full range of contrast.

#### The shape of the CRF depends predominantly on the inhibitory feedback

Since 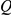 as defined by Eqn. (10) only depends on the strengths of the feedback connections from the inhibitory cells, 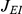 and 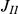, and not on the connections from the excitatory ones, 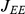 and 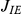, only modification of the first two can change the CRF shape from sub- to supersaturating.

Figs. 2A–C show the effect of changing 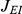 on the shape of the response of excitatory neurons when 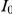 is increased. These responses increases monotonically and mildly saturate when 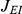 is small and displays supersaturation when 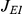 is large. This is because 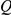 increases with 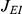.

**Figure 3:**
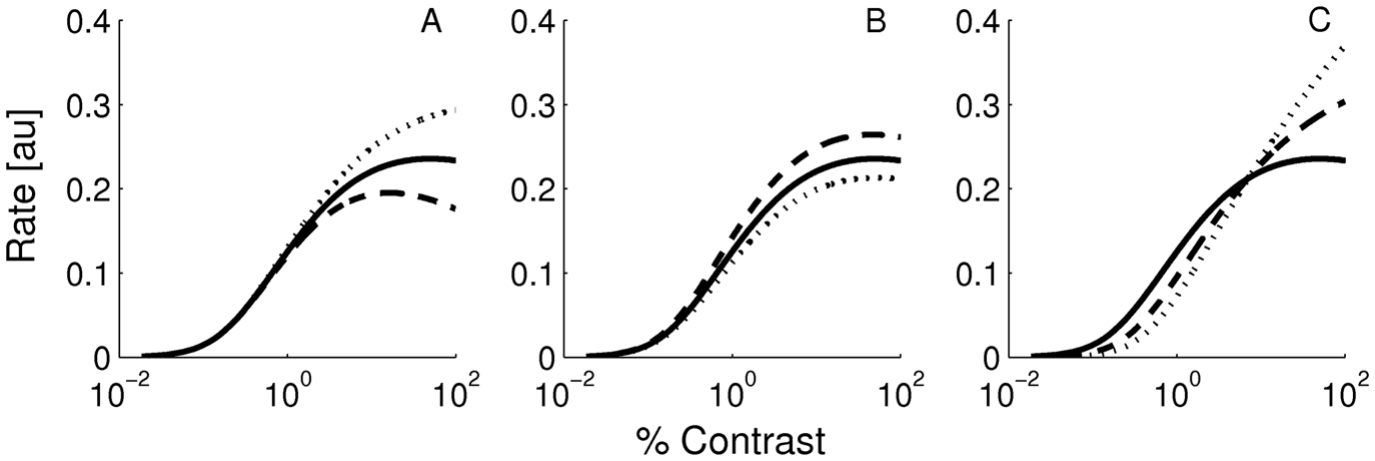
Dependence on network parameters of the CRF for the excitatory neurons. Solid lines: same parameters as in Fig. 1. A: change of inhibition to inhibitory coupling 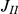 by 5%. Dashed line : 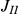 = 4.1, dotted line: 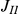 = 4.5. B: change of recurrent excitation 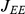 by 20%. Dashed line: 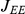 = 1.2; dotted line: 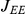 = 0.8. C: Change of exponent of the power-law 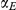. Dashed line: 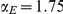 (dashed line); dotted line 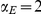.

The dependency of the CRF shape on 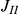 is depicted in Fig. 3A. For large 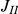, 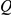 is small. Therefore, in that case the CRF increases monotonously with 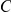. In contrast, for small 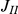, 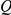 is large. Thus in that case we expect the CRF to be non-monotonic and to display super-saturation. Intuitively, it stems from the fact that the saturation of the CRF of the excitatory neurons is due to the inhibitory feedback they receive. As 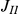 increases, this feedback decreases and this is more pronounced at high contrast, where 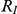 is large, than at low contrast.

The recurrent excitation, 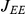, affects the shape of the CRF much less than inhibition, although it strongly affects the level of activity at large contrast. As shown in Fig. 3B, the effect of changing 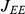 is roughly multiplicative. This holds in a wide range of changes in 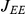 as large as 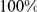 (not illustrated).

#### Dependence of the CRF on the power-law exponents of the neuronal transfer functions

Equation (10) predicts that, at high contrast, 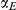 and 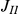 affect the CRF of the excitatory neurons in a qualitatively similar way. This is illustrated in Fig. 3C where CRFs are plotted for three values of 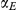, while keeping 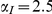. Only the CRF for the smallest value of 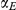 displays saturation at large contrast. This is because as 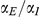 increases, the inhibitory feedback is reduced at high contrasts. This is expressed by the fact that 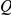 is proportional to the ratio 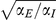.

### Response properties of the neurons in the conductance based model

In this section, we investigate to what extent the results we have obtained in our simplified rate model still hold in a more realistic conductance-based model, in which neuronal dynamics is governed by voltage-dependent conductance channels and synaptic interactions are mediated by conductance changes (see Eqn. 23). We also investigate in this framework how far diversity in the intrinsic cell properties or in the connectivity can account for the heterogeneity in the CRFs observed experimentally (present study and [1]–[6], [8]).

#### The input-output transfer functions of the neurons in the conductance-based model

**Figure 4:**
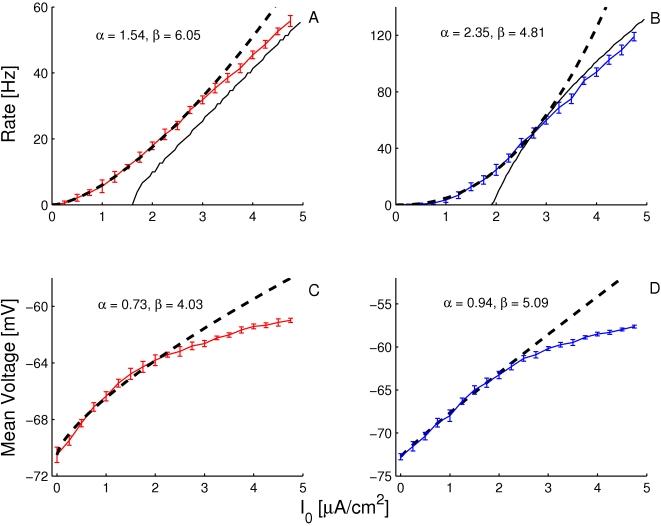
The 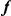-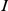 (A,B) and the 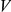-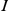 (C,D) transfer functions of the neurons in the conductance-based model. Left: excitatory neurons. Right: inhibitory neurons. Black solid lines (A,B): transfer functions in the absence of noise. Blue/Red solid lines: transfer functions in presence of noise (parameters given in Table 3). Dashed lines: power-law fit. The activity and the sub-threshold voltages (clipping the spikes at −50 mV) were averaged over a time window of 1.5 sec and 10 repetitions. Error bars correspond to the error on the mean.

The frequency-current (f-I) transfer functions of our conductance-based excitatory and inhibitory model neurons are plotted in Fig. 4A,B. In the absence of noise (solid lines), the firing rate increases as a square root near current threshold (as it is typical for type I neurons, [58]), and linearly beyond. Note that the excitatory neurons have a smaller gain than the inhibitory ones. This is mainly due to the level of adaptation, which is larger in the excitatory neurons than in the inhibitory neurons (note that activity was averaged over a time window of 1.5 sec). This adaptation linearizes the f-I curve of the excitatory neurons [58].

The conductance-based network model is studied below for a fixed level of input noise, chosen so that the standard deviation of the sub-threshold membrane potential fluctuations is on the order of 3–4 mV, as measured *in-vivo* [23]. The f-I curves with such a noise level have a convex shape (Fig. 4A,B), which can be well fitted with a power law [46]. The parameters of this fit depend on the range of currents over which one makes it. The dashed lines (Fig. 4A,B) correspond to the best fits in the range 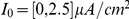 (or equivalently, firing rates in the ranges [0, 30] Hz and [0, 60] Hz for excitatory and inhibitory neurons respectively). The exponent of the power law is 2.35 for the inhibitory neurons and 1.54 for the excitatory ones.

The bottom panels in Fig. 4 show the V-I curves for the excitatory (Fig. 4C) and inhibitory (Fig. 4D) neurons. Fitting these curves with a power law in the same range of external inputs (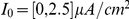) gives exponents smaller than 1, (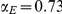, 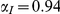).

#### Transfer function for synaptic inputs

The effect of a synapse on the dynamics of a neuron consists of the injection of a time-dependent current combined with a time-dependent increase in the neuron input conductance. It is not obvious how the rate model developed in the previous section can account for the combination of these effects. Shriki et al. [59] addressed a similar issue in the case where the noise in the network is weak and the input-output transduction function of the neurons is well approximated by a threshold linear function. They showed that the shunting effect of the synaptic conductances can be accounted to a good approximation by a shift of the neuronal transduction function. This shift is proportional to the conductance change, provided the neurons fire sufficiently asynchronously and the external inputs vary sufficiently slowly. Consequently, the stationary properties of the conductance based network can be obtained by solving the mean field equations for an effective rate model.

**Figure 5:**
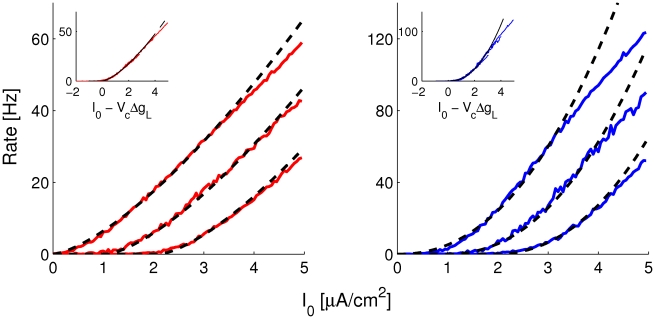
Transfer functions of excitatory (left panel) and inhibitory (right panel) CBM neurons in presence of noise and different values of 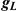. From left to right, 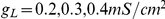. Solid lines: simulation results. Dashed lines: best fits to the power-law function: 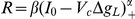 with 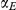 = 1.44, 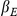 = 6.46, and 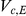 = 10.57 and 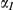 = 2.23 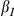 = 5.19, and 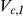 = 9.45, for all 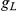. Insets: same data as a function of 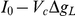.

If the background noise is large, the shunting effect due to the synapses can affect the gain 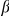 as well as the exponent 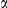 of the effective power-law transfer function. To generalize the approach of Shriki et al. to the situation of our model, we studied the f-I curves of the neurons in the presence of noise (the same amount as in Fig. 4) for different values of the leak conductance 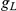. The results are depicted in Fig. 5. This shows that increasing 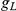 translates the f-I curves to the right, effectively shifting the threshold by an amount that, in the range of 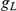 we explored, is approximately linear in 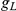. In contrast, the gain, 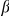, and the exponent, 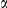, of the power law are not sensitive to 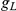. Indeed, the best fits of the f-I curves obtained in intervals of currents of similar amplitudes for different values of 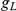 superimpose well once normalized for the shift in threshold (insets in Fig. 5). Note however that as 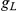 increases, the range in firing rates in which the fit is good becomes smaller.

### Orientation selectivity and contrast-response functions in the conductance-base model

We simulated a network model of V1 made of these conductance-based neurons. The effect of a visual stimulus is modeled by adding an input 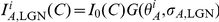 to the neurons. We take the connection widths such that they satisfy the condition: 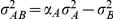 (see Eqns. (7, 8)) where 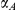 are given by the best fit of the f-I curves (see above and Fig. 5). The maximal LGN input, 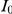, depends on the contrast 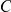:
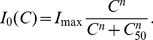
(11)The width of the LGN input to the excitatory and inhibitory populations is 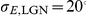 and 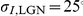 respectively.

**Figure 6:**
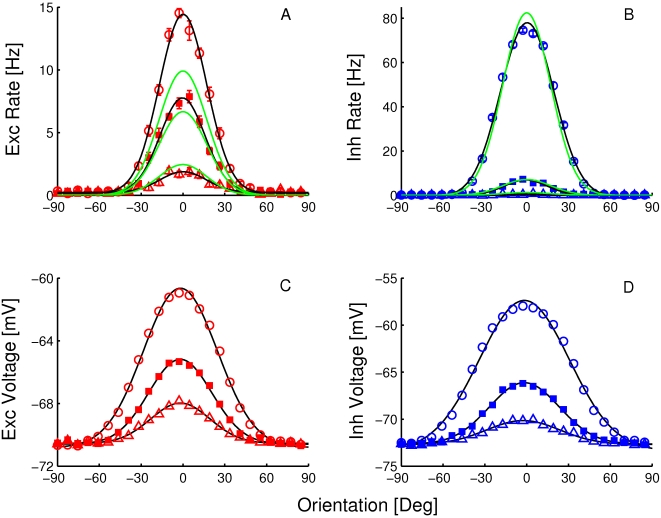
Orientation tuning curves for the spike response (A: excitatory neurons, B: inhibitory neurons) and voltage (C: excitatory neurons, D: inhibitory neurons). Triangles: 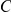 = 3%, squares: 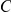 = 6%, circles: 25%. Green lines in A and B are the predictions from the effective rate model. Black solid curves are fits to a Gaussian.

Fig. 6 shows the orientation tuning curves for the firing rate and average voltage of both neuron types. The firing rate tuning curve is well fitted by a Gaussian for both types of neurons. The width of the optimal Gaussian changes by less than 10% when the contrast increases from 1 to 64%. For this contrast range, the effective leak conductance, 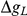, increases from 0 to 0.19 mS/cm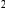 for the excitatory neurons and from 0 to 0.13 mS/cm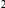 for the inhibitory ones.

We have also plotted in Fig. 6 the predictions given by the effective rate model. For the excitatory population, the simulations results differ substantially from the prediction of the rate model; the rate model underestimates the peak of the tuning curve of the excitatory neurons by as much as 30% (Fig. 6A). The discrepancy is less substantial for the inhibitory population (Fig. 6B). It may be surprising that the discrepancy is larger for the excitatory neurons than for the inhibitory neurons, whereas the deviations from a power-law in Fig. 5 is *bigger* for the former than for the later. However, this can be explained as follows.

According to Fig. 5, the inhibitory rate should be lower in the spiking network than in the effective rate model. This, however, also decreases the inhibitory feedback to the 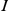 population. This decreased inhibitory feedback cancels the effect of the deviation from power law of the f-I curve to a large extent. For the excitatory neurons the fit to a power-law is good for the whole input range, but the 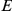 population also receives less inhibitory feedback than predicted from the effective rate model. This leads to a substantial increase in the firing rate of the excitatory neurons, compared to what one would expect from the effective rate model (Fig. 6A).

In contrast to the height of the tuning curves, there is surprisingly little discrepancy between the numerical simulations and the predictions of the effective rate model for what concerns the width of the tuning curves. This also stems from the corrective effect of the inhibitory feedback. The inhibitory feedback to the inhibitory populations suppresses the broadening of the output tuning curve implied by the deviation of the power-law. As a result, the width of the inhibitory feedback to the excitatory cells is close to that predicted by the effective rate model. Hence the excitatory tuning width is also close to the predicted one.

Because in the CBM the average voltage varies almost linearly with the input, the tuning curve of the voltage follows the tuning curve of the net input. Since the latter is close to a Gaussian with a contrast independent width, the voltage tuning curves are well approximated by Gaussians and have a close to contrast-invariant tuning width, as shown in Fig. 6C,D. Note that voltage tuning width is substantially broader than the tuning width of the spike response.

#### The CRFs of V1 neurons saturate before the input CRF

**Figure 7:**
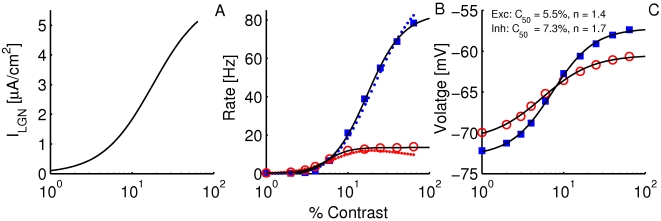
CRFs in the conductance based model. A: CRF of the LGN input, 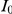, in the excitatory and inhibitory neurons (Eqn. (11), with 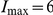 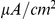, 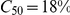 and 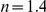). B: CRF of the spike response. Circles: excitatory neurons, squares: inhibitory neurons. Solid lines: best fit to H-ratio function. Parameters of the fits: 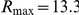 Hz, 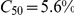, and 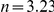 for the 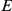 population. 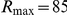 Hz, 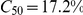 and 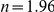 for the 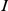 population. Dotted lines: prediction of the effective rate model. C: CRFs of the voltage. Circles: excitatory neurons, squares: inhibitory neurons. Solid lines: best fit to H-ratio functions.

The CRFs of the LGN input is plotted in Fig. 7A and the CRFs for the spike response of the excitatory and inhibitory neurons are displayed in 7B. The latter CRFs are well fitted to an H-ratio function. The CRF of the inhibitory neurons has almost the same 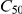 as the LGN input (18% and 17.2%, respectively). In contrast, the CRF of the excitatory neurons is steeper than the LGN input CRF (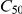 = 5.6%). This is similar to what was observed in our rate model and this is due to the sharp increase in inhibition from the network at high contrast.

Fig. 7C shows the CRFs of the voltages. These are also well fitted by an H-ratio function. In both excitatory and inhibitory neurons, the voltage CRFs saturate approximately as early as the excitatory spike rate, reflecting the effect of excitatory recurrent input on the voltage contrast-saturation. The exponents 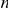 of the voltage CRFs, however, are smaller than those of the spike rate. This is because for the spike rate, the exponent 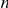 is significantly affected by the nonlinearity of the f-I curve.

#### Sensitivity to changes of synaptic width

**Figure 8:**
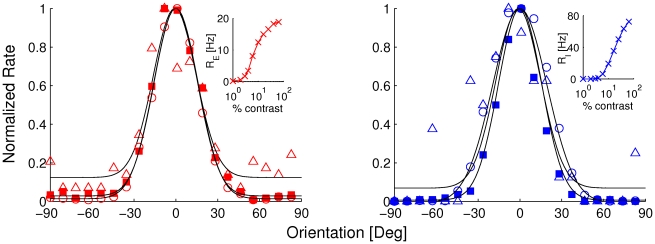
Orientation tuning curves of normalized responses for an excitatory neuron (left) and an inhibitory neuron (right), when 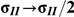. Visual stimuli of 3% contrast (triangles), 6% contrast (squared), and 25% contrast (circles). Solid curves are fits to a Gaussian function. 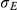 at low, medium and high contrasts is: 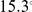, 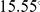, and 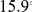 respectively. 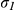 is: 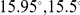, and 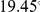 respectively. In the interval of contrast [1%,64%] the tuning widths are 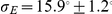, and 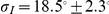 ( mean 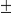 SD). Insets: the CRF of the excitatory and inhibitory neuron.

The theory predicts contrast invariance of the tuning if the width of the synaptic feedback tuning satisfies Eqns. (7) and (8). It is unlikely that these conditions are exactly satisfied. Therefore it was important to check that contrast invariance is not sensitive to deviations from these conditions. This robustness is depicted in Fig. 8 where the normalized tuning curves of an excitatory and an inhibitory neuron are plotted for a value of 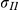 reduced by a factor of 2. Even with this drastic deviation from Eqns. (7, 8) the tuning width of the excitatory and inhibitory neurons change by less than 10% and 15% respectively when the contrast is increased from 1% to 64%. The sensitivity to changes in the width of the other feedback connections is even less (not shown). Thus the mechanism does not require precise fine-tuning of the feedback width.

### Diversity of the CRF shapes

Fits to the H-ratio function of the CRFs of V1 neurons reveal a large diversity in the parameters 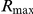, 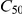 and 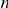 [1]–[6], [8]. Can this diversity be accounted for in the framework of our model ?

#### Heterogeneity in synaptic inputs

We investigate first how heterogeneities in the synaptic inputs to the neurons contribute to the diversity of their CRFs. Heterogeneity in the inputs originates in the retina or in the LGN, where CRFs differ significantly between cells [3], [9]–[12], [14]. At the same time, a proportion of single cells in V1 combine inputs from LGN cells with distinct properties [4], [60]. Alternatively, recurrent interactions within V1, or feedback inputs from areas higher in the visual pathway, could contribute to this variability.

The number of recurrent connections a given neuron in V1 receives is on the order of several thousand. Therefore, one expects that the fluctuations in the recurrent synaptic input will be much smaller than its average. Therefore, these fluctuations should contribute only weakly to the CRFs diversity, unless they are correlated, or the network is in a state of balance of excitation and inhibition (see Discussion).

A more significant contribution to this diversity is expected from the variability in the inputs from the LGN, which are much less numerous; it has recently been estimated that the firing of a spike in a cortical simple cell results from the functional convergence of 30 LGN cells only [61].

In our conductance based model the LGN input is 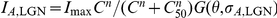. We incorporated variability in this input by randomly selecting, for each neuron, the parameters 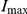, 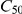 and 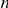 from flat distributions. Specifically, we took 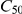 uniformly between 8% and 28% and 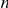 between 1 and 1.8. These values are in agreement with experimental data for magnocellular neurons [3]. We further assumed that 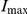 is distributed between 3.5 and 4.5 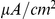.

**Figure 9:**
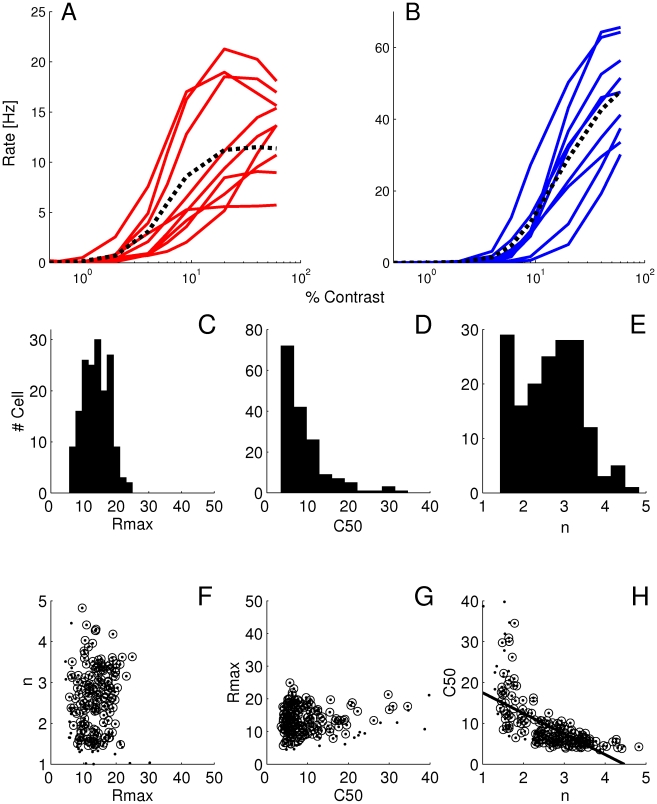
Diversity in CRFs induced by heterogeneity of the LGN input. Examples of CRFs of excitatory (A) and inhibitory (B) neurons. Dotted lines: population averaged CRF. Histograms of the H-ratio fit parameters 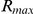 (C), 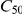 (D) and 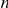 (E) for all the excitatory neurons. Bottom: pair-wise scatter plot of these parameters, 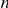 vs. 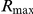 (F), 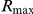 vs. 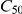 (G) and 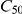 vs. 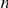 (H). Dots in the scatter plot show all neurons. Circles shows neurons with a good fit (N = 326). The correlation coefficient between 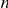 and 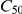 is 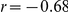. The other two correlations are not statistically significant.

Our simulations show that heterogeneity in the LGN input generates diversity in CRF shapes of the V1 neurons (Fig. 9A,B). Since the heterogeneity in the total feedback input 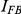 is small (see above), the CRF of neuron 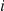 can be approximated by 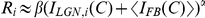. Therefore, depending on the LGN input a neuron receives, its CRF can super-saturate or semi-saturate. For example, if the 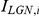 saturates at high contrast, then the increase in 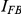, which is negative, will lead to super-saturation. Since on average the inhibitory activity does not saturate, inhibitory neurons do not show super-saturation.

Despite the presence of heterogeneities, the CRFs could be well-fitted with an H-ratio function for most neurons (84%) in our model network. The distribution histograms of the 3 parameters of the fit are shown in Fig. 9C–E for the excitatory neurons. The distributions are broad, 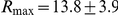 Hz, 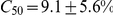 and 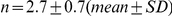, with dispersion comparable to that observed experimentally. Broad distributions are also found for the CRF parameters of the inhibitory neurons in our network with 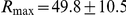 Hz, 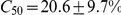 and, 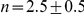 (not illustrated).

We next examined correlations between the CRFs parameters values. Neurons with a bad fit, (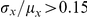 for 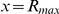, 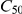 or 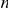) were excluded from this analysis. We found a negative correlation between the best fit estimates for 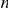 and 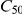 across the excitatory neurons population (Fig. 9H). This can be explained as follows: on the one hand, variability in 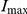 has only a weak effect on the response to low contrast of the neurons. Thus the contrast at which the neuron's response starts to increase significantly from baseline is relatively unaffected by the heterogeneity of the synaptic input. On the other hand, the larger 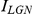, the higher the contrast has to be before the inhibitory feedback becomes large enough to induce a saturation in that neuron's response. This both increases the range of contrast over which he output varies and hence *decreases* the parameter 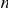. Since 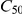 is approximately halfway between the contrast at which the response starts and the point of saturation, it also *increases* 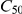. Therefore the correlation between 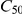 and 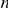 will be negative. In contrast, 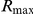 is not significantly correlated with 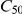 and 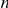 (Fig. 9F,G).

#### Heterogeneity in single neuron properties

Another possible source of CRFs diversity is the heterogeneity in intrinsic properties of V1 neurons. To investigate this contribution, we now assume that all the neurons receive the same LGN input, with parameters 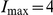 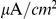, 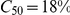, and 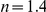, but now ascribe heterogeneous parameters for single neuron dynamics. Specifically, we assume that the adaptation conductances, 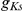, are uniformly distributed over the range [0.5,6.5] mS/cm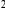 and [0.1,1.1] mS/cm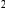, for the excitatory and the inhibitory neurons, respectively, resulting in variable adaptation strength for V1 neurons, as reported experimentally [62], [63]. As a consequence, 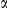, the exponent of the effective input-output function of the neurons, varies from neuron to neuron. We also introduce heterogeneities in the leak reversal potential which is uniformly distributed between −77.5 mV and −67.5 mV. This is equivalent to an effective heterogeneity in the spike threshold current of the neurons, 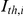, as reported experimentally [30], [64], [65]. Therefore, the CRF for neuron 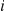 can be written as 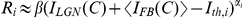.

**Figure 10:**
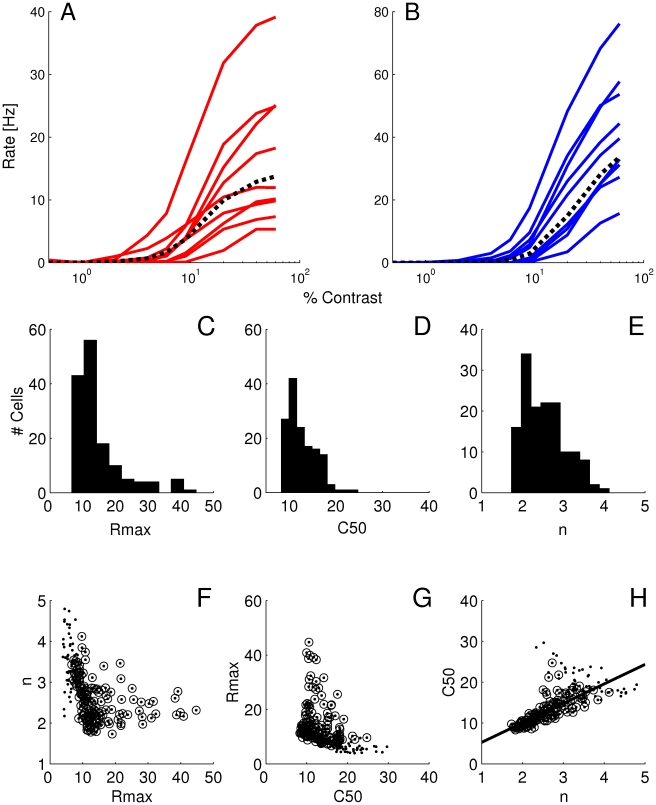
Distribution of CRFs induced by heterogeneous neuron properties. Examples of CRFs for (A) excitatory and (B) inhibitory neurons. Dotted lines: Population averaged CRF. Distribution histograms of the the H-ratio parameters 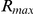, 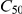 and 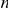 are shown for excitatory neurons in C, D and E respectively (all the neurons are included). Bottom: pair-wise scatter plot of these parameters, 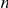 vs. 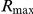 (F), 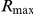 vs. 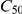 (G) and 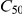 vs. 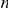 (H). Dots in the scatter plot show all neurons, and circles show neurons with a good fit (N = 368). The correlation coefficient between 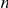 and 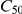 is 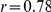. The other two correlations are not statistically significant.

Examples of CRFs obtained under these conditions are given in Fig. 10A,B. As in the case of LGN input heterogeneities, the excitatory CRFs are steeper (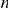 is higher) and saturate earlier (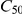 is lower) than in the LGN inputs. Here also, most of the CRFs are well fitted to the H-ratio function (92%). However, none of the cells exhibit super-saturation. This is because 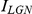 and 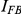 are the same for all the neurons. The heterogeneity in 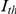 and 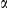 can only shift and scale the CRF.

The distributions of the parameters of the H-ratio function which fit the CRFs are broad (Fig. 10C–E). We found that 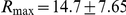 Hz, 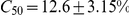, 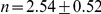 and 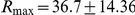 Hz, 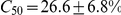, 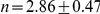, for the excitatory and the inhibitory neurons, respectively.

We examined correlations between the CRFs parameters values, considering only the neurons for which the CRF was well fitted by an H-ratio function. The correlations between 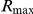 and the other two parameters of the CRF are not significant (see Fig. 10F,G), as is the case when the heterogeneity is due to LGN input diversity. However, now a positive correlation exists between 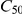 and 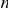. This can be understood as follows: since all neurons receive the same synaptic input, the contrast at which the response saturates is roughly the same for all cells. However, the variability in spiking threshold has a major effect on the contrast at which the neurons begin to respond. Neurons with a low threshold start firing at lower contrasts, and therefore will have a larger contrast range over which the output varies, and hence 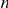 will be smaller. But because they start to fire earlier, 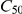 will also be smaller for these neurons compared to cells with higher spike threshold. As a result, 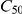 and 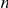 are positively correlated.

#### Combination of LGN input and single neuron heterogeneities

It is quite likely that, in reality, heterogeneities both in LGN input and in neuronal properties contribute to the diversity of CRFs shape in V1. The results we have just described suggest that these contributions would hardly be disentangled by relying solely on the shape of the distribution histograms for the parameters 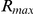, 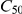 and 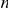. As a matter of fact, the shape of the distributions are very similar, whether the heterogeneities are in the LGN inputs or in the neuronal properties. However, the *sign* of the correlation between the parameters 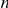 and 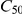 is different in the two cases. This suggests that it may be possible to quantify the contribution of these two sources of heterogeneity by examining this correlation.

To combine both sources of heterogeneities, we took distributions of LGN and single neuron parameters with the same shapes and same means as above, but with widths narrowed by a factor 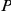 and 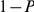 respectively. Thus, when 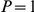, we only have heterogeneities in the LGN input, while if 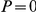, there are only heterogeneities in the intrinsic properties of the neurons.

**Figure 11:**
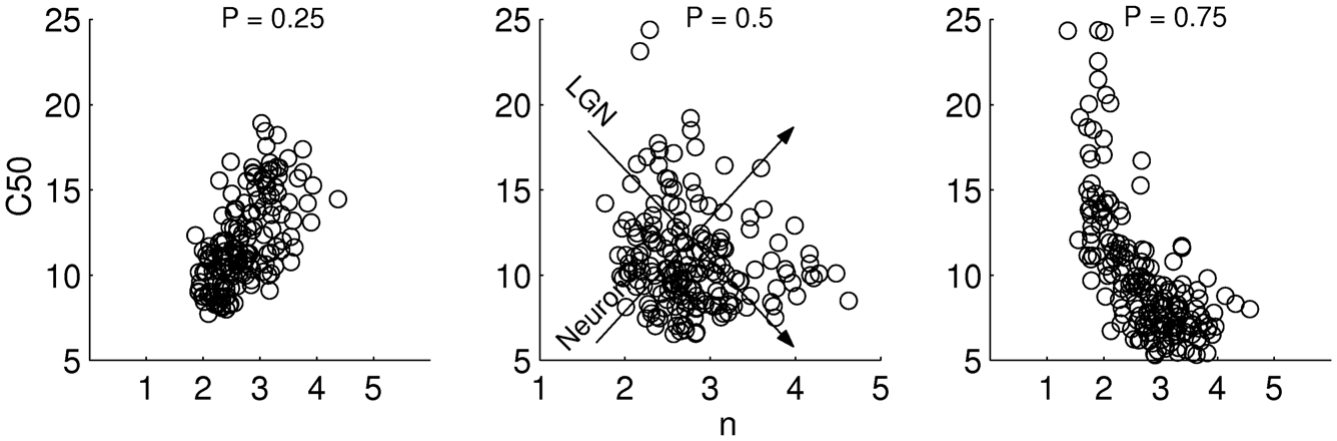
Scatter plot of 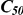 and 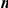 for heterogeneities in both LGN input and single neuron properties (excitatory neurons only). Correlation between 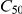 and 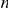 decreases from positive to negative as the diversity due to single cell properties is decreased and the diversity due to the LGN input is increased.

We performed numerical simulations of the network with different values of 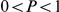. For each value, we computed the parameters of the CRFs fit for all the neurons. In Fig. 11, 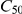 is plotted vs. 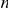 for all the excitatory neurons which have a CRF well fitted by an H-ratio function and for three values of 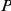. This shows, as expected, that as 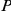 increases from 0 to 1, the correlation changes from positive to negative. For 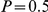, there is an approximate balance between the effects of the heterogeneities in the LGN input and those in the cell properties and the correlation is small.

**Figure 12:**
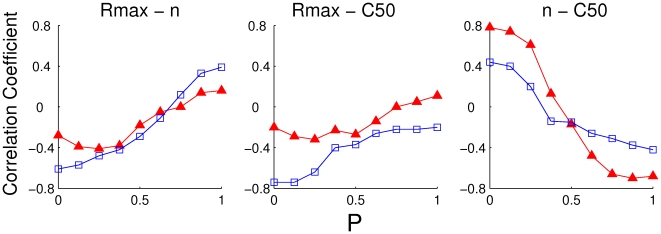
The correlation coefficient values for all parameters for excitatory (triangles) and inhibitory neurons (squares). Except for P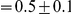, the absolute value of the correlation coefficient for inhibitory neurons is higher than for the excitatory ones for the correlation between 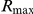 and 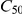 or the exponent 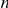. For the correlation between 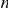 and 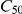 the excitatory neurons show stronger correlations than the inhibitory ones.

The correlation coefficients for 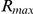 vs. 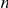, 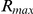 vs. 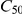, and 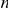 vs. 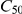 are plotted as a function 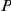 in Fig. 12 for both excitatory and inhibitory neurons. When considering 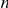 vs. 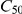, the correlation appears in general weaker for the inhibitory population than for the excitatory population. Except for the range P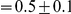, there should be clear differences between the two neuron types. However, for both populations, the sign of the correlation changes around 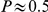. As a consequence, a crossing between the two lines occurs around that value of 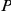. Thus, correlations are significantly reduced for both cell types only when LGN input and intrinsic neuronal properties contribute equally to CRFs diversity.

#### Comparison with experimental data

To get an insight on the contribution of the different sources of heterogeneity in reality, we examined CRFs for extracellularly recorded neurons in the primary visual cortex of anesthetized marmoset monkeys. Stimuli were drifting gratings presented with the orientation and spatial frequency optimal for the cell under study. The CRFs were produced using 12 contrasts values between 2 and 90%. The data were fitted with an H-ratio function of the form 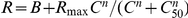.

**Figure 13:**
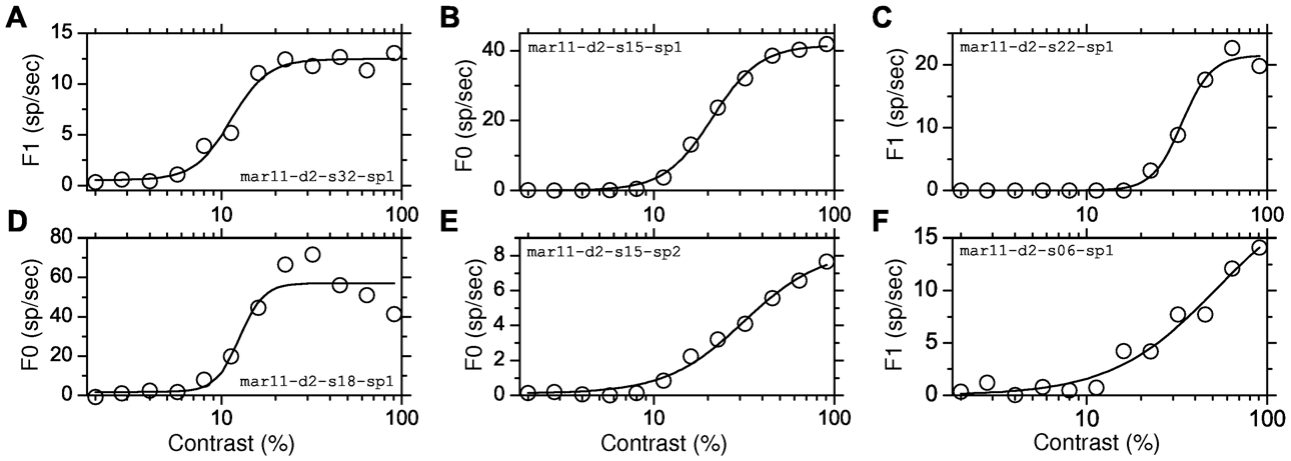
Examples illustrating the variety of CRFs calculated for single neurons in marmoset V1. Continuous lines represent the H-ratio function fitted to the experimental data (open dots). The y-axis corresponds to the firing rate modulation F1 for simple cells (A, C, F) and to the F0 component for complex cells (B, D, E). The figure depicts examples of saturating CRFs (A,B) mildly saturating CRF (C), and strongly supersaturating CRFs (D). Non-saturating CRFs are plotted in E and F. Parameters of the H-ratio fits are: A: 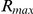 = 11.9 sp/sec, 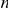 = 4.28, 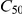 = 11.3%. B: 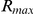 = 41.6 sp/sec, 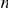 = 3.28, 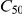 = 21.0%. C: 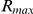 = 21.5 sp/sec, 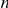 = 5.32, 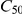 = 33.7%. D: 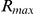 = 55.4 sp/sec, 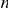 = 6.49, 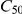 = 12.6%. E: 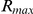 = 8.2 sp/sec, 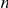 = 2.01, 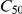 = 31.5%. F: 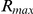 = 20.4 sp/sec, 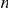 = 1.49, 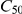 = 52.6%. The 6 examples presented here were obtained during one single electrode penetration in one marmoset; Therefore the diversity illustrated here is not due to inter-individual variability.

Fig. 13 shows some examples. CRFs were established using the F0 component in complex cells and the F1 component in simple cells. There was no significant difference between simple and complex cells for any of the parameters of H-ratio function. Simple and complex cells have therefore not been distinguished in the population data analysis.

When classified as described in the Methods, saturating cells represented 53 of the 98 cells (Fig. 13, A,B), non-saturating cells 25 (Fig. 13, E, F), and super-saturating ones 20 cells (Fig. 13, C, D), in proportion similar to the one reported by Peirce [8] in macaque V1.

**Figure 14:**
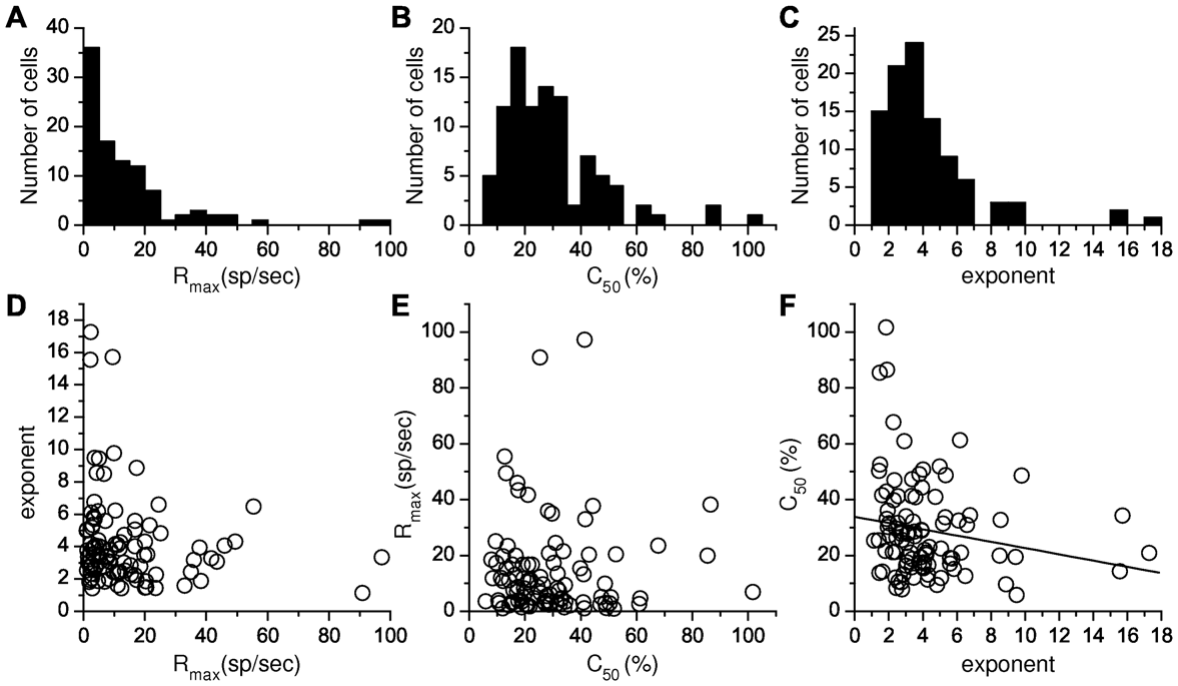
Distributions and correlations between CRF parameters obtained in marmoset V1. A. 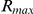. B. 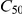 C. The exponent 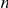. D. Panels (D) and (E) show that there is no significant correlation between 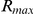 and 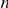 and between 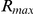 and 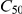. Panel F shows that correlation between 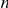 and 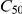 is on the margin of significance (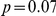 and 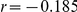 with Fisher's test, but 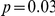 and 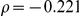 with the non parametric Spearman rank correlation). The line represents the linear correlation.

In our sample of 98 cells, the median 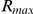 was 8.7 sp/sec (interquartile: 14.0), the median 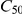 was 25.5% (interquartile: 17.2) and the median exponent was 3.44 (interquartile: 2.54). Distributions for the exponent and 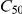 (Fig. 14) appear comparable to those obtained in macaque V1 [3]. Both appear to be distinct from those measured in either the magno- or parvocellular layers of the macaque LGN [3]. It is also to be noticed that the proportion of cells in our database displaying saturating or super-saturating response is much larger than in marmoset LGN [14], [66], [67].

Qualitatively, the presence of super-saturating cells in V1 would support the “heterogeneous LGN inputs” model, as super-saturation does not occur in the “heterogeneous intrinsic properties” model. To refine this conclusion, we examined the correlation between CRF slope and 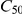. The results were on the margin of significance: using Fisher's test (a test that supposes an affine relationship between variables), the 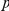 value was 0.07 and the correlation coefficient, 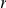, was −0.185. On the other hand, a non parametric test (Spearman rank correlation) returned a significant correlation with a 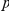 value of 0.03 and a correlation coefficient, 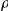, of −0.221. These results suggest that heterogeneities in LGN inputs and heterogeneity in neuronal intrinsic properties both contribute to the diversity of CRFs in V1, with a possible slightly greater contribution for LGN inputs heterogeneity.

## Discussion

Many theoretical studies have previously investigated possible mechanisms explaining the contrast invariance of the width of the orientation tuning curves measured in neurons in primary visual cortex [15], [23], [33]–[37], [42]–[44], [46], [47], [49]–[51], [54]. Some studies have provided theoretical explanations for the contrast-response functions of these neurons [42], [48]–[50], [52], [53]. However, only a few of them have examined both features together, either unsuccessfully [42] or using parameter regimes that may not be relevant to the *in vivo* situation (membrane time constants: [49], [50]; synaptic depressions: [52], [53]).

### The transfer function

All the findings of the present paper rely on the fact that, in the presence of noise, the effective input-output transfer function is accelerating and can be fitted by a power-law over the physiological range of neuronal responses to visual stimuli [15], [23], [46], [47], [68], [69]. The noise in the input influences the neuron's transfer function by effectively smoothing the effect of the spiking threshold. The mean input current and voltage are also non-linearly related, such that the rate-voltage transfer function is well fitted by a power-law, but with an exponent that is larger than the one of the rate-current transfer function. In the present model, the exponent, 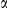, of the input-output transfer functions of the neurons must be larger than 1 to insure spike tuning curves sharper than the tuning curves of the LGN input. For neurons *in vivo*, the transfer function for voltage vs. firing rate is well approximated by a power-law, with an exponent, ranging between 2 and 5 [15], [23], [68].

Under the assumption that the input noise is on the same order for different neuron types, the input-output transfer function of our model inhibitory neurons accelerate more than that of excitatory neurons. This is because inhibitory neurons have higher gain and show less firing rate adaptation (e. g., [63], [70]). Thus, the fit of the spiking rate to a power-law reveals different exponents 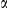, for the different neuron types in our model. That the exponent tends to be higher in the inhibitory cells than in the excitatory ones has been reported in recent experimental studies [69].

A major difference between the rate model and the conductance-based model is that, in the later, synaptic inputs increase the effective leak conductance, an effect that was not taken into account in the former. Nevertheless, we have shown here that an increase, 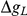, of the leak conductance, if not too large (increasing the effective 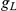 up a factor of 2) has the same effect on the transfer function as an additional negative current, 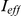. This current is proportional to 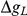, 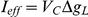. This is similar to what was found by Shriki et al. [59] for the transfer of conductance based neurons in the absence of noise. As we have shown, this allows for the derivation of an effective rate model, which replicates the steady state behavior of the CBM.

### Role of the feedforward and feedback inputs: Contrast invariance of orientation tuning

Noise, as inferred from voltage traces, has been reported to be independent of stimuli contrast and orientation [23] (but see [15], [71]). Such a noise in the input current effectively results in a power law transfer function [46], [47]. It has been shown that, in the absence of recurrent cortical interactions and with feedforward inputs alone, the power-law transfer function leads to an approximate contrast invariance of the orientation tuning curve width [46], given contrast invariant input width, as they emerge from the spatial arrangement of LGN ON and OFF cells [17], [61]. Due to the nonlinearity of the transfer function the outputs are more tuned than the inputs by the factor 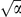. Here we extended these results to take into account recurrent cortical interactions. We showed that they remain true provided that the synaptic distributions have an appropriate spatial extent, namely that the conditions expressed by Eqns. (7, 8) are satisfied.

When the conditions for the width of the feedback, expressed by Eqns. (7, 8) are satisfied, the feedback interactions do not contribute to the sharpening of the tuning. The latter is determined by the tuning of the LGN input, together with the sharpening effect of the power-law transfer function. This is in sharp contrast to the role of recurrent interactions in network models of V1 studied previously [33]–[37], [72]. Recurrent interactions, however, appear essential for explaining the shape of the CRFs (see below).

### Role of the feedforward and feedback inputs: Contrast-response function

In the absence of recurrent interactions, the CRF of the cortical neurons is shifted toward higher contrast compared to the CRF of their feedforward inputs. This means that to achieve a reasonably large response at low contrast the parameter 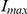 of the LGN input must be quite large. This implies that, at maximum contrast, the response of the cortical neurons is large too. However, beyond a critical value, the response amplitude would fall in a range where the transfer function of the neurons deviates substantially from a power-law. In our conductance-based model, this deviation becomes appreciable above 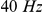. In turn, this deviation from power-law implies substantial deviations from contrast-invariance of the tuning-width at high contrast. Therefore, the strong inhibitory feedback in the recurrent network model we have studied plays a crucial role, which is to regulate the high contrast responses, relative to the responses at intermediate and low contrast. As a result, both feedforward and excitatory recurrent inputs can be relatively strong, resulting in a consistent response for both low and intermediate contrast, yet the response at high contrast does not reach values beyond which contrast invariance is lost. We have demonstrated this role in our conductance-based model. The saturation due to the feedback which keeps the response within the power-law range for high contrast also causes a decrease of the 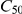 and an increase in the slope of the CRF relative to the LGN input.

### Tuning of the LGN input

We have modeled the LGN input as a Gaussian, with a width that is independent of contrast. This represents a simplification, which is nevertheless justified given previous theoretical studies on contrast invariance of orientation tuning in simple cells.

A well known problem in this context [31], [43] is that, in simple cells, the LGN input generates an untuned DC component in the membrane potential response, which grows faster with contrast that the tuned AC component. A solution to this problem consists in canceling this DC component by including either anti-phase or broadly tuned inhibition in the models [43], [45], [72]. This was not explicitly incorporated in our model. We rather simplified it with a tuned LGN input that one should view as a *net* input into the cells which combines both the actual LGN input and the feedforward inhibition.

### Comparison with experimental data

The conditions expressed by Eqns. (7, 8) imply specific range for the synaptic connections between sub-populations of neurons. They show that, if the orientation tuning width of inhibitory neurons is broader than that of excitatory neurons as reported experimentally [28], [30] the synaptic projection from inhibitory to excitatory neurons should be narrower than the projection width from excitatory to excitatory neurons. This is compatible with anatomical data, which show that the spatial extent of inhibitory connections is usually less than that of excitatory connections [73], [74]. Note that these conditions were obtained under the assumptions of Gaussian inputs and outputs, which are in line with experimental data (e. g., [75]).

Here an important caveat should be made. We showed that contrast invariance of the tuning width is robust to violations of conditions Eqns. (7, 8). If the range of the synaptic feedback, both excitatory and inhibitory, is changed by as much as 50%, contrast invariance is still nearly achieved with a relative error of less than 10%. Thus the model predictions about the relative extent of the excitatory and inhibitory feedback should not necessarily be taken as quantitative.

The parameters we used generated relatively narrow tuning curves (see Results), in accordance with the tuning width reported for layer 4 simple cells in some studies (e. g., [15], [69]). However, others studies reported a large heterogeneity of tuning width, including broadly tuned cells and cells showing a non-negligible response at the orthogonal orientation [7], [19], [20], [28], [30], [76]. We therefore checked whether our results were valid for parameter regimes different from the one we initially used. We simulated networks with broader tuning curves (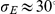), for which the response at the orthogonal orientation was approximately one tenth of that at the preferred orientation. For such networks, we found that the orientation tuning width did not change significantly with contrast. However, the ratio of the response at the orthogonal orientation versus the preferred orientation decreased slightly with contrast. Interestingly, this departure from strict contrast-invariant orientation tuning has been observed experimentally for broadly tuned cells in some studies [7], [19]; but see [20]. However, this should not be taken too seriously because, as Fig. 8 shows, deviations from Eqns. (7, 8) for the feedback width can have a substantial effect on the response at the orthogonal orientation, which could result in the reverse effect.

### The origins of the diversity in the CRF of V1 neurons

The CRF of the spike response can be well fitted by an H-ratio function in a large fraction of V1 neurons. However, the parameters of the function are highly diverse across neurons [1]–[4], [7]. Most studies that aim to explain contrast invariance or the shape of the CRF ignore this heterogeneity and usually do not indicate whether the proposed mechanism can accommodate a large diversity of responses.

Whether the excitatory neurons saturate or not is determined by the strength of the feedback connections, particularly from the inhibitory cells. This implies that some degree of fine-tuning of these strengths is necessary if we impose that the average excitatory CRF saturates at 100% contrast. Because of this sensitivity, relatively small variability in the feedback strengths for individual neurons leads to rather large changes in the CRFs. This can contribute to the large variability in CRFs, with non-saturating, saturating and super-saturating cells observed in the primary visual cortex of the same animal.

Here we have investigated other possible sources for this diversity, focusing on the contribution of variability in single neuron intrinsic properties, and on the contribution of heterogeneities in the CRFs of LGN neurons. We have demonstrated that these two sources of variability can both account for the diversity observed in experiments. In addition, our model predicts a correlation between the parameters 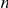 and 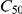, which is either negative or positive, depending on the source of heterogeneities. The strength of the correlation is further predicted to be reduced when both sources are mixed, in proportion to the relative contribution of each.

We examined CRFs for neurons in the primary visual cortex of marmoset monkeys. The parameters 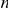 and 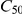 obtained in these experimental data were at best weakly negatively correlated. This suggests that heterogeneity in the LGN input may contribute slightly more than the neurons' intrinsic properties to the diversity of CRFs shape.

Another possible source of heterogeneity we did not examine is heterogeneity in the recurrent feedback inputs. We assumed that these are uncorrelated. Then, given their large number comparatively to LGN inputs, heterogeneities in feedback inputs would cancel each others and this would result in an “averaged” CRF input to all neurons. However, some studies showed that subset of excitatory and inhibitory neurons may form specific connections with other neurons [77]–[80], and in many cases the connections are not reciprocal. This would lead to heterogeneity in the feedback input, that we expect to have the same effect on the correlations between 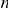 and 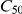 as the diversity in the feedforward input. Other studies [81], [82], however, suggest that inhibitory fast spiking cells establish a dense network with other neurons, as assumed in the present study.

### Perspectives

Two major weaknesses of our model is that we have to add *external* noise to the system to obtain voltage fluctuations that are biologically plausible and that it does not exhibit heterogeneity in the orientation tuning curves.

One way to obtain input fluctuations *intrinsically* is to use a model that operates in the balanced regime [83], [84]. In this regime, heterogeneity in the response naturally arises from the strongly amplified effect of randomness in the connectivity. However, in their current formulation, balanced network models cannot explain the shape of the CRF as observed experimentally. This is because in such networks the population averaged response should scale linearly with the external input [83], [84], so that on average the 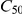 of both the excitatory and inhibitory populations should be the same as the 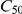 of the LGN input, in contrast to what is observed experimentally. It is our hope that development of such models, in which recurrent connections are responsible for the synaptic noise which is so essential to contrast-invariance of tuning width, will help further integration of feedforward and feedback models for a better understanding of the mechanisms at work in cortical processing.

## Materials and Methods

### Ethics statement

The protocol for the experiments which are reported here is in accordance with guidelines of the French ministry of agriculture (décret 87/848) and the European Union (directive 87/609).

### The rate based model

Our rate model consists of 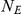 excitatory and 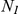 inhibitory neurons. The firing rate of excitatory neuron 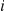 and inhibitory neuron 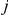, denoted by 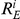 and 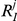 respectively satisfy
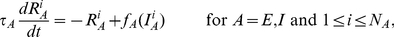
(12)where 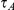 is the membrane time constant for population 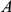, 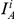 is the total, noise averaged, input into the neuron, and 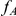 is the effective, noise averaged, transfer function. Following recent experiments [23], [68] and theoretical studies [46], [47], we assume that the transfer function 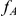 is a threshold power-law function, 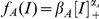. Here 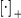 denotes the half rectified linear function, 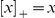 for 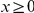 and 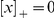 for 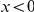. The exponent of the power law function is 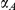 and 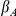 sets its scale.

Our model network represent a hypercolumn in V1 and has the geometry of a ring [33]. Neuron 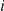 in population 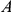 is characterized by an angle 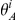, defined as the orientation of the visual stimulus for which the LGN input it receives is maximum. We model this input as
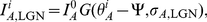
(13)where 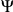 is the orientation of the stimulus, 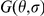 is the 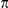-periodic Gaussian with width 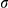, defined as 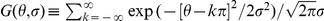. 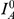 gives the overall strength of the LGN input and depends on the stimulus contrast. As we will see, for 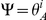, not only the LGN input to neuron 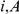 is maximum but so is also of its spike response. Therefore, 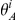 is also the preferred orientation of the neuron.

We assume that 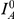 varies with the contrast, 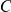, of the visual stimulus as 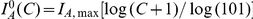 where 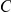 is in percents. This logarithmic dependence, which does not saturate, was chosen to facilitate the analysis of the cortical network.

The preferred orientations of the neurons are uniformly distributed over the interval 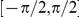. The feedback input from the network to neuron 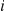, 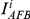, is given by
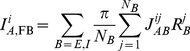
(14)where the synaptic strengths, 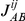, depend on the difference in preferred orientations between neurons 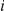 and 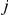 and falls off with this difference as a periodic Gaussian with width 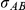


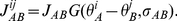
(15)Note that we have scaled the synaptic strength by the density of neurons. The number of neurons in population 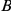 with preferred orientation between 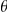 and 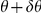 is equal to 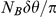, which explains the factor 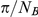 in Eqn. (14).

In the limit of large 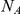, we can replace 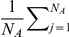 by 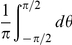, and 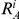 by 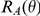, where 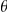 is a continuous variable. The rates 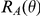 satisfy the dynamics
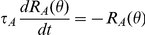


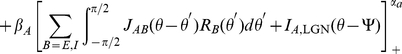
(16)Due to the rotation symmetry of the network, the response 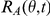 of the neurons depends on the stimulus orientation 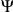 only through the difference, 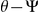, between this orientation and the neurons preferred orientation, 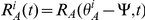. Thus we need only to consider the case where 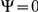.

#### Steady states

In the steady state we have 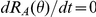, so that:
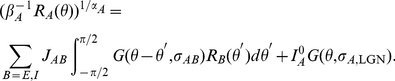
(17)A general analytical solution to this set of integral equations is not available. However, as we show in the Results, if 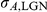 is much smaller than 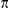 and the connection widths 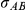 satisfy 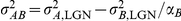, the firing rates are approximately given by
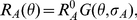
(18)where 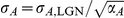 and 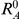 is given by
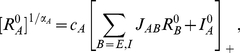
(19)with 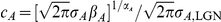.

#### Stability analysis

The stability of the steady state, 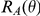 is investigated by setting 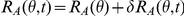, where 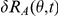 is a small perturbation. Linearizing the dynamics, Eqn. (16), around the steady state one obtains that the perturbations satisfy
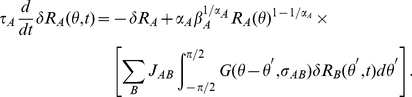
(20)This system has a discrete eigenvalue spectrum and the 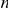
th eigenmode, 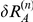, satisfies 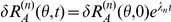, where 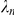 is the eigenvalue associated with this eigenmode. If the real part of 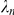 is negative the eigenmodes decay to zero. Any perturbation 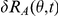 can be written as 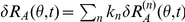 with some constants 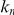. If all the eigenvalues 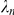 have negative real part, so that all 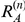 decay to zero, every small perturbation decays and hence the steady state is stable.

### The conductance based model

In the conductance-based network, neurons are point-like and the dynamics of their membrane potential, 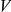, is:
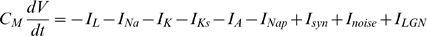
(21)where 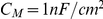. The first term on the right-hand side of Eqn. (21) is the leak current 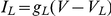. The next five terms correspond to a sodium current, 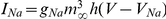, a delayed rectifier potassium current, 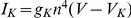, responsible for the up and down-stroke of the action potential respectively, a slow potassium current, 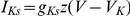, inducing spike adaptation, an A-type potassium current, 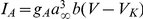, which becomes active during the hyper-polarization period and affects the length of the inter-spike interval, and a persistent sodium current, 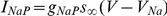, which tends to amplify small depolarizations.

The gating variables 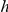, 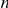, 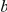, 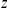 follow the dynamics:
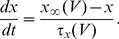
(22)For 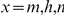, the functions 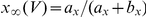 and 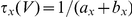, with the parameters 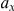 and 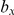 as given in Table 1 and for 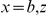 the functions 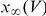 and 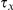 are given in Table 2. The maximal conductances of the ionic channels of the excitatory and inhibitory neurons are given in Table 3. They are chosen to reproduce qualitatively the frequency-current transfer functions of regular spiking excitatory neurons and fast spiking inhibitory neurons, such that excitatory neurons have a lower threshold [85] and stronger spike frequency adaptation than inhibitory neurons (e.g., [63], [70]).

**Table 1:** Gating variable of the conductance-based model.

x	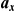	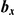
m	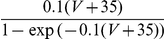	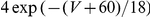
h	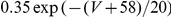	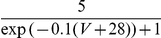
n	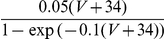	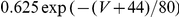

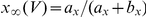
 and 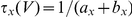 (in msec).

**Table 2:** Gating variables of the ionic channels in the conductance-based model.

x	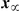	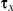
a	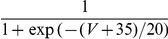	NA
b	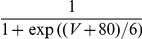	20
s	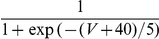	NA
z	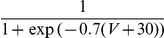	50

NA = not applicable; 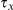 in msec.

**Table 3:** Conductance density in mS/cm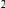 and the reversal potentials in mV for the ionic channels of a excitatory (E) and inhibitory (I) neurons in the conductance-based model.

	E	I
x	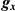	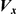	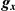	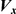
L	0.2	−70	0.2	−70
Na	35	55	35	55
NaP	0.12	55	0.08	55
K	15	−90	7.5	−90
A	2.5	−90	7.5	−90
Ks	2.5	−90	0.25	−90

The terms left on the right-hand side of Eqn. (21) are the synaptic inputs, 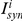, the neuron receives because its recurrent interactions with the other neurons in the network, a current, 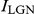, representing the feedforward inputs from the LGN to V1, and the noise 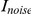.

The synaptic current received by neuron 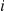 in population 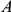, is
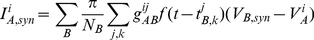
(23)where 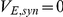 mV and 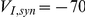 mV are the reversal potentials of excitatory and inhibitory synapses respectively. The strength of a synapse connecting the presynaptic neuron 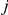 in population 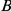, to postsynaptic neuron 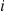 in population 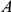, is characterized by 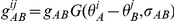, where 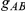 is given by Table 4. Note the normalization to the neuronal density 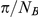. The term 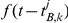 describes the contribution of the 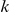
th spike of neuron 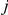 in population 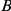, which occurred at time 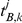, to the synaptic conductance at time 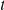. We take
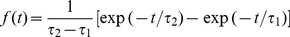
(24)with rise time constant 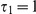 msec and decay time constant 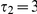 msec for excitatory as well as for inhibitory synapses. The current, 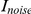, is a Gaussian white noise with zero mean. Its standard deviation, 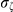, is chosen such that the standard deviation of the membrane potential of the neurons is approximately 3–4 mV, as measured experimentally in V1 [23].

**Table 4:** Synaptic conductance density in mS

msec/cm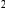 for the conductance based model network.

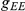	1
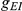	4
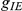	1
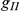	2.6

The LGN input is modeled as in Eqn. (13) with 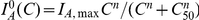, where the values of 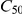 and 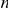 are taken in accordance with experimental data for magnocellular cells [3] and 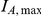 is such that the activity of the neurons are similar to those measured in V1 during visual stimulation [3], [6].

#### Effective rate model

In our CBM network, because feedback is generated through synaptic conductances, the effective membrane conductance of the neurons depends on the network state. When the latter is stationary, the recurrent feedback induces an increase of this conductance which is equivalent to an increase in the leak conductance of the neurons. Shriki *et al.* [59] have shown that in absence of external noise, a change by a small amount, 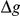, affects the frequency-current transduction function of the neurons as if a current, proportional to 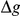, 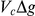, was subtracted from its input. Using single neuron simulations we show, in the Results, that this is still the case if the neurons receive a stationary input with noise, provided that the firing rates are not too high. This allows us to formulate an effective rate model for the conductance based network as follows: the total input to neuron 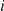 of population 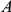


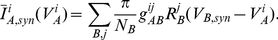
(25)can be decomposed into 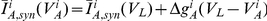, where 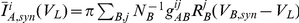 is the current into the neuron at rest and 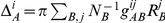 is the effective increase in leak conductance. Thus, if with an extra leak 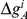 the input-output relation of the neuron is given by 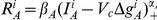, we obtain that at equilibrium
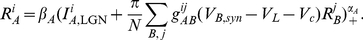
(26)This equation is the same as the fixed point equation of the rate based model if we set 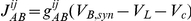.

### Numerical simulations and analysis of the results

In the rate model we simulated networks with 100 neurons for each of the populations, using a second order Runge Kutta integration scheme with a time step of 1 msec. After verifying that this discretization was sufficiently fine, we used these simulations to find the fixed points in the rate equations and to verify the stability of steady state.

The conductance-based model dynamics of networks consisting of 400 excitatory and 400 inhibitory neurons was simulated using a second order Runge-Kutta integration scheme with a time step 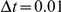 msec. For each contrast, ten trials with different noise realizations were simulated and the responses were averaged over a time window of 1.5 sec after elimination of a transient.

The orientation tuning curves of the neurons were fitted with Gaussians parametrized as: 
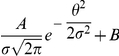
. For the rate model, we set the offset, 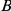, to zero. For the CBM, 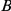 was in general non-zero because the noise induced a non-zero activity at cross-orientation. The peak amplitude of these Gaussians estimated for different contrast, 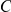, yielded the CRFs of the neurons, which were subsequently fitted with the H-ratio function [1]:
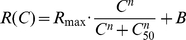
(27)where 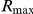 is the maximum firing rate, 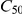 is the contrast (in 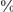) for 
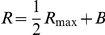
 and the exponent, 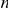, is a measurement of the function's steepness. In the case of the CBM, we additionally computed the relative error of the estimated values of the CRF parameters (the relative error on 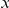 is its SD divided by its mean, 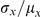). Good fits were defined as those with relative errors smaller than 0.15 for all the parameters.

### Experimental data

Experimental data for the CRF was obtained from marmoset monkeys (Callithrix Jacchus, 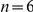). Details about the experimental protocol can be found in [20]. One half hour before anesthesia induction, the animals were tranquilized with diazepam (Valium, Roche) (i. m., 3 mg/kg) and atropine (0.05 mg/kg) was given at the same time to reduce secretions and to prevent bradycardia. Anesthesia was induced with Alphadalone/Alphaxalone acetate (Saffan, Essex Pharma, 1.2 ml/kg) injected intramuscularly and maintained during surgery by i. v. injection (0.17 ml/kg every 10–15 minutes). Synthetic corticoids were given to prevent brain edema. Animal's body temperature was maintained at 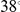
C using a heating pad controlled by a rectal thermistor. EKG recording was performed through metallic pliers.

The surgical procedure consisted first in placing a catheter in the femoral vein. Next, a tracheotomy was performed to allow artificial ventilation. The marmoset was then set in a stereotaxic frame. Two holes were drilled over the frontal cortex and Ag wires inserted for epidural EEG recording. A craniotomy was made to gain access to area V1. A head post was sealed to the skull and fixed to the stereotaxic apparatus.

Following surgery, the animal was artificially ventilated with 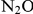/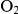 (50%/50%). Anesthesia and analgesia were supplemented by a continuous infusion of sufentanil citrate (Sufenta, Janssen, 4–6 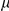
g/kg/hr) after a loading dose of 1 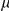
g/kg. The infusion vehicle was made of the mixture of 2 ml glucose 30%, 15 ml of amino-acid perfusion solution (Totamin, Baxter) and included synthetic corticoids (0.4 mg/kg/hr); NaCl was added to a final volume of 50 ml. We waited for 1–2 hours of infusion with this solution to ensure adequate depth of anesthesia. The animal was then paralyzed by adding pancuronium bromide (Pavulon, Organon, 0.1 mg/kg/hr) to the solution described above. Mydriasis and cycloplegia were induced with ophthalmic atropine sulfate (1%, Alcon). Gas permeable contact lenses were used to protect the eyes. The heart rate, rectal temperature and expiratory 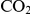 concentration were monitored throughout the experiment and maintained at 250–350 bpm, 37–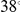
C and 3–5%, respectively. The EEG and the absence of reaction to noxious stimuli were regularly checked.

Action-potentials were recorded extracellularly in area V1 using tungsten-in-glass microelectrodes. Spike-sorting was performed using Spike2 (Cambridge Electronic Design, Cambridge, UK) system. Appropriateness of single-unit isolation was based on the refractory period of the neuron. Visual stimuli were presented onto a computer monitor placed at 114 cm from the animal's eyes. We first determined the preferred orientation using square-wave drifting gratings. Optimal spatial frequency was then determined using sine-wave drifting grating. The CRF was then established using sine-wave drifting grating with optimal orientation and spatial frequency, presented at 12 different levels of contrast increasing geometrically for 2 to 90%. All visual stimuli were presented in a circular patch of 2–6 degrees diameter, centered on the receptive field. Drift velocity was between 0.5 and 2 cycles/sec. To avoid transient responses, the contrast was incremented in a 1 sec duration ramp, maintained at steady level for 3 or 4 sec, then decreased back to 0% in a 1 sec duration ramp, then maintained at 0% contrast for 1 sec. The measurement of mean firing rates was restricted to the 3–4 sec plateau period. The fits of the CRF to a H-ratio function was performed as with the simulations data (see above). The quality of the fit was good, (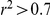) except one supersaturating cell (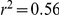) but there was no good reason to exclude this cell. The mean 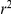 was 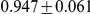 (S.D.) and the median 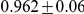 (interquartile).

Receptive fields were classified as “simple” or “complex” on the basis of the relative modulation (F1/F0 [86]) in their response to gratings at the optimal spatial frequency. In our data set, the distribution of F1/F0 was bimodal, with a gap at 1. Cells were considered as simple when the relative modulation was 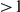 and complex when it was 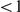 [86].

A cell was considered to display saturating response when the response extrapolated to 100% contrast was equal to 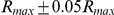. It was considered as non-saturating when the extrapolated response was less than 0.95 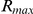 and as super-saturating if the response to at least one of the test contrast below 90% was larger than 1.05 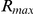.

## Supporting Information

Text S1 Supporting information.
(0.15 MB PDF)
