# Supplementary material for: Power-Law Input-Output Transfer Functions Explain the Contrast-Response and Tuning Properties of Neurons in Visual Cortex
Source: PLoS Comput Biol. 2011 Feb 24;7(2):e1001078. doi: 10.1371/journal.pcbi.1001078 (PMC3044767; doi:10.1371/journal.pcbi.1001078)
Supplement: Text S1 — Supporting information. (0.15 MB PDF) [file pcbi.1001078.s001.pdf]

Supplement to Power-Law Input-Output Transfer Functions Explain the Contrast-Response and Tuning Properties of Neurons in Visual Cortex

Erez Persi[1], David Hansel[1,2], Lionel Nowak[3], Pascal Barone[3], and Carl van Vreeswijk[1*]

[1]Laboratoire de Neurophysique et Physiologie
Université Paris Descartes, Paris, France.
[2]Interdisciplinary Center for Neural Computation, The Hebrew University, Jerusalem, Israel.
[3]Cerco, Université Toulouse 3, CNRS, Toulouse, France.

A. Derivation of Eigenvalues with Hermite Integrals

We consider the perturbed firing rates, $R_A+\delta R_A$, where R_A is the steady state solution. Linearizing the rate dynamics in δR_A we obtain for the dynamics of the perturbations

$$\tau_A \frac{\partial \delta R_A(\theta,t)}{\partial t} = -\delta R_A(\theta,t) + \alpha_A R_A^{1-1/\alpha_A} \left[\sum_B \int_{-\pi/2}^{\pi/2} d\theta' J_{AB}(\theta-\theta') \delta R_B(\theta',t) \right]. \quad (1)$$

Since this is a linear set of equations, perturbations can be decomposed into eigen-modes $\delta R_A^{(n)}(\theta)$, with eigenvalues λ_n, in the following way

$$\delta R_A(\theta,t) = \sum_{n=0}^{\infty} C_n \delta R_A^{(n)}(\theta) e^{\lambda_n t} \quad (2)$$

*corresponding author, email: cornelis.van-vreeswijk@parisdescartes.fr

where C_n are some constants.

Finding an analytical expression for the eigen-modes and eigenvalues for this system appears to be impossible. But if $\sigma_A \ll \pi$ we can approximate the periodic Gaussians by normal Gaussians and take the integral from $-\infty$ to ∞ in stead of from $-\pi/2$ to $+\pi/2$. If we make this approximation $\delta R_A^{(n)}$ satisfies

$$(\tau_A \lambda_A + 1)\delta R_A^{(n)}(\theta) = \alpha_A \left(\frac{R_A^0 e^{-\theta^2/2\sigma_A^2}}{\sqrt{2\pi}\sigma_A} \right)^{1-1/\alpha_A} \left[\sum_B \frac{J_{AB}}{\sqrt{2\pi}\sigma_{AB}} \int_{-\infty}^{\infty} d\theta' e^{-(\theta-\theta')^2/2\sigma_{AB}^2} \delta R_B^{(n)}(\theta') \right] \quad (3)$$

Because the right hand side of this equation involves the convolution of $\delta R_B^{(n)}$ and a Gaussian it is useful to write the eigen modes in terms of Hermite functions. We thus write the eigen-modes as

$$\delta R_A^{(n)}(\theta) = \sum_{k=0}^{\infty} a_A^{n,k} H_k(\sqrt{\epsilon_A}\theta) \cdot e^{-\epsilon_A \theta^2} \quad (4)$$

where ϵ_A is a scaling factor which we will choose later so as to make the expansion in Hermite functions as simple as possible.

Inserting Eqn 4 into 3 we obtain

$$\sum_{k=0}^{\infty} (\tau_E \lambda_n + 1) a_A^{n,k} H_k(\sqrt{\epsilon_A}\theta) e^{-\epsilon_A \theta^2} = \alpha_A \left(\frac{R_A^0}{\sqrt{2\pi}\sigma_A} \right)^{1-1/\alpha_E} \times$$
$$\times \left[\sum_{B,k} \frac{J_{AB}}{\sqrt{2\pi}\sigma_{AB}} a_B^{n,k} \int d\theta' e^{-\frac{(\alpha_A-1)\theta^2}{2\alpha_A \sigma_A^2} - \frac{(\theta-\theta')^2}{2\sigma_{AB}^2} - \epsilon_B \theta'^2} H_k(\sqrt{\epsilon_B}\theta') \right]. \quad (5)$$

We multiply both sides by $H_p(\sqrt{\epsilon_E}\theta)$ and integrate over θ. Using the orthogonality of the Hermite polynomials, $\int_{-\infty}^{\infty} d\theta H_n(\theta) H_m(\theta) e^{-\theta^2} \neq 0$ only if $m = n$, we obtain

$$(\tau_E \lambda_n + 1) a_A^{n,p} = \sum_{B,k} M_{AB}^{k,p} a_B^{n,k}, \quad (6)$$

where

$$M_{AB}^{k,p} \propto \int d\theta' \int d\theta \; e^{-\frac{(\alpha_A-1)\theta^2}{2\alpha_A \sigma_A^2} - \frac{(\theta-\theta')^2}{2\sigma_{AB}^2} - \epsilon_B \theta'^2} H_k(\sqrt{\epsilon_B}\theta') H_p(\sqrt{\epsilon_A}\theta)$$
$$= \int d\theta e^{-f\theta^2} H_p(\sqrt{\epsilon_A}\theta) \int d\theta' \cdot e^{-\frac{1}{\Sigma}\left(\theta' - \frac{\theta}{g}\right)^2} H_k(\sqrt{\epsilon_E}\theta'), \quad (7)$$

where $f = (\frac{\alpha_A - 1}{2\alpha_A \sigma_A^2} + \frac{\epsilon_B}{g})$, $g = (1 + 2\epsilon_B \sigma_{AB}^2)$ and, $\Sigma = 2\sigma_{AB}^2/g$.

Using $\int dx e^{-(x-y)^2} H_k(x+y) = H_k(y)$, this can be written as

$$M_{AB}^{k,p} \propto (\frac{1}{g})^{n/2} \int d\theta e^{-f\theta^2} H_p(\sqrt{\epsilon_A}\theta) H_k(\sqrt{\epsilon_B/g}\theta). \tag{8}$$

This integral is simple to solve if $\epsilon_A = 1/2\sigma_A^2$. In this case $f = 1/2\sigma_A^2$, so that we can use the result for Hermite polynomials

$$\int H_k(\gamma x) H_p(x) e^{-x^2} = 0, \tag{9}$$

except

$$\int dx H_{2m+p}(\gamma x) \cdot H_p(x) e^{-x^2} = \sqrt{\pi} \frac{(2m+p)!}{m!} 2^p \gamma^p (\gamma^2 - 1)^m, \tag{10}$$

for $m = 0, 1, 2, \ldots$.

This leads to the upper-triangle structure of the eigenvalues matrix

$$\left(\begin{array}{ccccc} M^{00} - D(\lambda) & 0 & M^{02} & 0 & \ldots \\ 0 & M^{11} - D(\lambda) & 0 & M^{13} & \ldots \\ 0 & 0 & M^{22} - D(\lambda) & 0 & \ldots \\ 0 & 0 & 0 & M^{33} - D(\lambda \ldots & \\ \vdots & \vdots & \vdots & vdots & \ddots \end{array} \right),$$

where M^{pk} and $D(\lambda)$ are the 2×2 matrices

$$M^{pk} = \left(\begin{array}{cc} M_{EE}^{pk} & M_{EI}^{pk} \\ M_{IE}^{pk} & M_{II}^{pk} \end{array} \right), \qquad \text{and} \qquad D(\lambda) = \left(\begin{array}{cc} \tau_E \lambda - 1 & 0 \\ 0 & \tau_I \lambda - 1 \end{array} \right) \tag{11}$$

respectively. The eigenvalues are given by the values of λ for which $\det(M^{kk} - D(\lambda)) = 0$. From this we see that the eigen-function for the nth mode has the form

$$\delta R_a^n(\theta) = \sum_{k=0}^{\lfloor \frac{n}{2} \rfloor} a_a^{n,n-2k} \cdot H_{n-2k}(\sqrt{\epsilon_a} \cdot \theta) \cdot e^{-\epsilon_a \theta^2}. \tag{12}$$

Thus, the first two modes involves only $H_0(x) = 1$, meaning that the perturbation of this mode is proportional to the steady state distribution. These modes reflect changes in amplitude of the rate profile. The next two modes involves only $H_1(x) = 2x$, thus these mode can be interpreted as a tendency

to shift the peak of the Gaussian steady-state solution. Modes five and six contain a linear combination of $H_0(x)$ and $H_2(x) = 4x^2 - 2$ so these modes tends to change both the amplitude and the width of the Gaussian solution. Higher modes are more complicated.

To determine the eigenvalues we use that M^{nn}_{AB} are given by

$$M^{nn}_{AB} = J_{AB} \left(\frac{R^0_A}{\sigma_a \sqrt{2\pi}} \right)^{\frac{\alpha_A - 1}{\alpha_A}} \left(\frac{1}{\alpha_A} \right)^{n-\frac{1}{2}} \left(\frac{\sigma_B}{\sigma_A} \right)^{n+1} \tag{13}$$

So that the eigenvalues fr the modes $2n$ and $2n+1$ are given by

$$\begin{aligned} (\tau_E \lambda + 1) a_E &= M^{nn}_{EE} a_E + M^{nn}_{EI} a_I \\ (\tau_I \lambda + 1) a_I &= M^{nn}_{IE} a_E + M^{nn}_{II} a_I. \end{aligned} \tag{14}$$

or $\lambda = \frac{-b_n \pm \sqrt{b_n^2 - 4\tau_E \tau_I c_n}}{2\tau_E \tau_I}$, where:

$$\begin{aligned} b_n &= \tau_E (1 + M^{nn}_{II}) + \tau_I (1 - M^{nn}_{EE}) \\ c_n &= (1 - M^{nn}_{EE})(1 + M^{nn}_{II}) + M^{nn}_{EI} M^{nn}_{IE} \end{aligned} \tag{15}$$

The conditions for which $Re(\lambda_n) < 0 \; \forall \; n$ are illustrated in Figure 1.

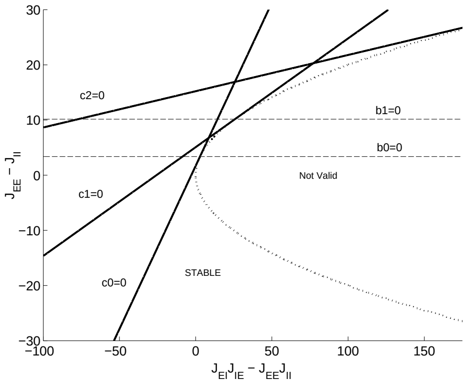

Figure 1: An example of a phase diagram in a symmetric case with $\alpha = 3$ and $\tau = 3$msec showing the stability of all modes in a large area of this plane. Solid lines: $c_n = 0$ for the first three modes. Dashed lines: $b_n = 0$, below which both eigenvalues of the mode n are negative. Doted line: separating curve for real synaptic matrix. Valid synaptic values are to the left of this curve. The 'triangle' formed between $c_0 = 0$, $b_0 = 0$ and the dotted curve contains the region where all modes are stable. See details in text.
